# Response to Letter to the editor on: “Never too late? Quadruplets at the age of 65 years”

**DOI:** 10.1007/s00404-021-06256-8

**Published:** 2021-11-10

**Authors:** Larry Hinkson, Christof Dame, Thorsten Braun, Irit Nachtigall, Wolfgang Henrich

**Affiliations:** 1 Department of Obstetrics, Charité–Universitätsmedizin Berlin, Chariteplatz 1, 10117 Berlin, Germany; 2 Department of Neonatology, Charité–Universitätsmedizin Berlin, 1335 Berlin, Germany; 3 Department of Preventive Medicine and Hygiene, HELIOS Hospital Bad Saarow, Bad Saarow, Germany; 4 Department of Anesthesiology and Operative Intensive Care Medicine (CCM, CVK), Charité–Universitätsmedizin Berlin, Berlin, Germany

Dear Editor,

We appreciate greatly the comments made by Rui-Hong Xue. Our report discusses an extreme and complex case of quadruplet pregnancy at a very advanced maternal age of 65 years with cross border in vitro fertilization (IVF) using donor eggs and sperms. As far as we are aware, it is indeed the most extreme case of multiple pregnancy in a postmenopausal woman following IVF published yet [1].

One of the issues that we addressed in the publication was the impact of women travelling across home country borders to access infertility treatments where the adherence to regulations from national authorities and recommendations from national or international professional societies governing infertility treatments are different, which may cause a lack of medical accountability. In this particular case, IVF was performed in Eastern Europe. Since some of the previous pregnancies of our patient (Gravida 14) were also induced by IVF at an age, when menopause is likely, the question on her ‘natural’ menopausal age cannot be reliably answered. Additionally, no information was provided on the treatment protocol, hormonal status or the number of embryos transferred. This in itself highlights a major issue as patients return to their home country for later pregnancy and delivery care without appropriate documentation of initial treatments. Thus, fertility specialists performing these procedures have a duty of care, that should not be abandoned as soon as the mother is pregnant [2].

Infertility treatment regulations are complex. In Germany, they are governed by national laws such as the Embryo Protection act which does not allow fertility treatments with donor eggs for IVF, a fact that is very controversially discussed [3]. Notably, the European Society of Human Reproduction and Embryology (ESHRE) provides a framework of good practice guidelines that should be followed where national laws are not established [4].

Confronted with the increased maternal and fetal risks such as in our case the option of unselected fetal reduction was indeed discussed. Of course we acknowledge the inherent moral and ethical challenges faced in such a situation which usually takes place between 12 to 14 weeks gestation [5]. Issues such as patient’s rights and autonomy, doctors duty of care and accountability, benevolence, non-maleficence and justice come into consideration. There is now growing evidence recommending the use of elective single embryo transfer (eSET) to limit the complex maternal and perinatal complications generated by multiple embryo transfer [6, 7]. For example, in Sweden with eSET rates of 82.5%, the rate of multiple birth is 3.1% as compared to 21.5% in Germany with eSET rates of 21.6% [8]. In our case, the decision declining eSET or unselected embryonic/fetal reduction was made by the physicians, who performed the embryo transfer, and the patient.

Despite the recommendations and guidelines from societies such as ESHRE, there seems to be no adherence to these consensus regulations in some countries. Unless regulations are enforced, there will occur extreme cases like ours in the future. The international IVF community and perhaps the World Health Organization (WHO) need urgently to find implementable solutions to protect mothers and children in every country, and also consider the fundamental right that children can obtain their true genetic descent at adolescent age.

## Data Availability

Data sharing is not applicable to this article as no datasets were generated or analyzed during the current study.
